# A Case of Primary Hyperparathyroidism due to Intrathyroidal Parathyroid Cyst

**DOI:** 10.1155/2014/213283

**Published:** 2014-12-29

**Authors:** Yavuz Yalcin, Turkan Mete, Recep Aktimur, Gultekin Ozan Kucuk, Gulhan Duman, Aysu Basak Ozbalci, Omer Alici

**Affiliations:** ^1^Department of Endocrinology and Metabolism, Ordu Training and Research Hospital, Ministry of Health, Ordu, Turkey; ^2^Department of Endocrinology and Metabolism, Samsun Training and Research Hospital, Ministry of Health, Samsun, Turkey; ^3^Department of General Surgery, Samsun Training and Research Hospital, Ministry of Health, Samsun, Turkey; ^4^Department of Radiology, Samsun Training and Research Hospital, Ministry of Health, Samsun, Turkey; ^5^Department of Pathology, Samsun Training and Research Hospital, Ministry of Health, Samsun, Turkey

## Abstract

Parathyroid cysts constitute 0.08–3.41% of all parathyroid masses. Intrathyroidal parathyroid cysts, however, are rare conditions with only a few cases being reported. Most of the parathyroid cysts are found to be nonfunctional and functional cysts are generally thought to be due to cystic degeneration of parathyroid adenomas. A cystic, smooth contoured lesion of 24 × 19 × 16 mm was observed in left thyroid lobe of a 76-year-old woman during ultrasonography which was performed as routine workup for primary hyperparathyroidism. It was defined as a cystic thyroid nodule at first. Tc^99m^ sestamibi scintigraphy was performed to see any parathyroid lesions, but no radioactive uptake was observed. Intact parathormone (iPTH) level was found to be >600 pg/mL in cyst aspiration fluid. Left lobectomy was performed, with a diagnosis of primary hyperparathyroidism due to functional parathyroid cyst. Serum iPTH level was decreased >50% postoperatively and histopathological evaluation was consistent with an encapsulated parathyroid adenoma with a cystic center. Parathyroid cysts are among rare causes of primary hyperparathyroidism. Diagnosis is made by markedly increased iPTH level in cyst fluid and observation of parathyroid epithelium lining the cyst wall.

## 1. Background

Primary hyperparathyroidism is not a rare disorder, with a yearly incidence of 21/1000000. Primary hyperparathyroidism is generally due to parathyroid adenoma and, less frequently, parathyroid hyperplasia, parathyroid cysts, and parathyroid cancer [1].

Parathyroid cyst may present as an asymptomatic neck mass or it may incidentally be detected during radiological evaluations or surgery for other reasons [2]. In more than 85% of cases, it is located in the neck and generally it originates from lower parathyroid glands [3]. Parathyroid cysts are generally nonfunctional. Nonfunctional cysts are seen more frequently in women and are detected as masses in the neck. Functional cysts are seen more frequently in men and they are secondary to degenerative changes in parathyroid tumor. Although many of the functional parathyroid cysts cause mild hypercalcemia, they may also present with acute parathyroid crisis symptoms [2, 4].

Parathyroid cysts may rarely be intrathyroidal and may mimic cold thyroid nodules [5, 6]. Aspiration of the cyst fluid may definitely diagnose parathyroid cyst. The diagnosis may be confirmed with parathormone (PTH) assessment in cyst fluid [2, 7]. In this study, we present a case of functional intrathyroidal parathyroid cyst which caused primary hyperparathyroidism.

## 2. Case

A 76-year-old female patient who was being followed due to operated bladder surgery was referred to Department of Endocrinology and Metabolic Diseases due to hypercalcemia and hypophosphatemia in routine evaluations.

**Figure 1:**
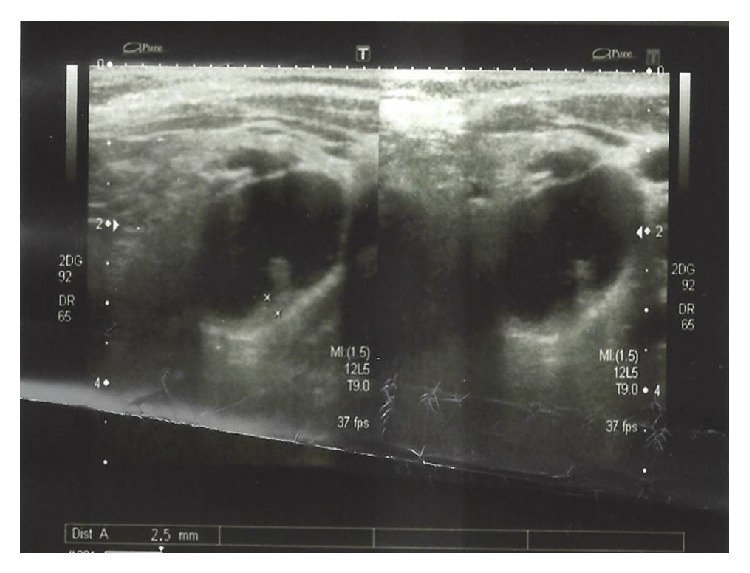
Ultrasonographic image of cystic lesion (17 × 26 mm) in left lobe of thyroid gland.

She complained of malaise and generalised bone pain and her physical examination revealed a 2 × 2 cm nodule in left thyroid lobe. Laboratory workup revealed hypercalcemia (12 mg/dL (reference range: 8,8–10,2 mg/dL)), hypophosphatemia (2,1 mg/dL (reference range: 2,5–4,5 mg/dL)), low 25 OH vitamin D (20,8 ng/mL (reference range: 10–70 ng/mL)), high intact parathyroid hormone (iPTH) (512,6 pg/mL (reference range: 13–92 pg/mL)), and normal thyroid function tests. Urinary calcium (Ca) excretion was found to be 3% in 24 hours. In thyroid sonography, parenchyma was mildly heterogenous, and there was a well contoured, 24 × 19 × 16 mm lesion at median posterior side of the left thyroid lobe which was mostly cystic in nature with a solid component towards the lumen at its posterior. It was considered as a cystic thyroid nodule (Figure 1).

**Figure 2:**
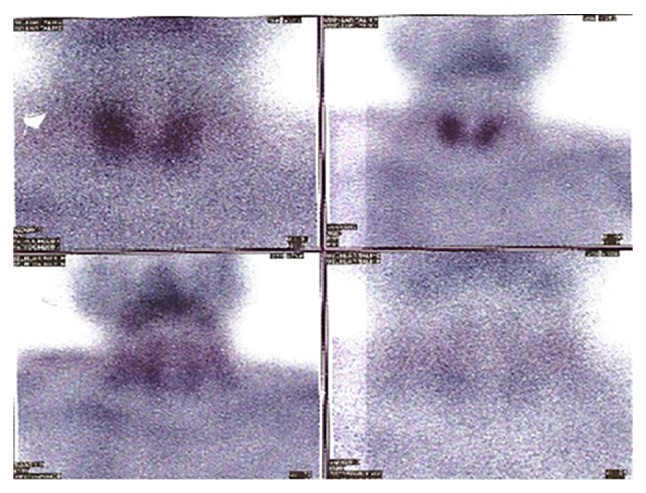
Tc^99m^ sestamibi scan showing radioactive contrast media excretion; no focal retention compatible with parathyroid adenoma/hyperplasia observed in neck or mediastinum.

In Tc^99m^ sestamibi scan which was performed to show parathyroid gland abnormality, no uptake of radioactive material was observed in the lesion (Figure 2).

**Figure 3:**
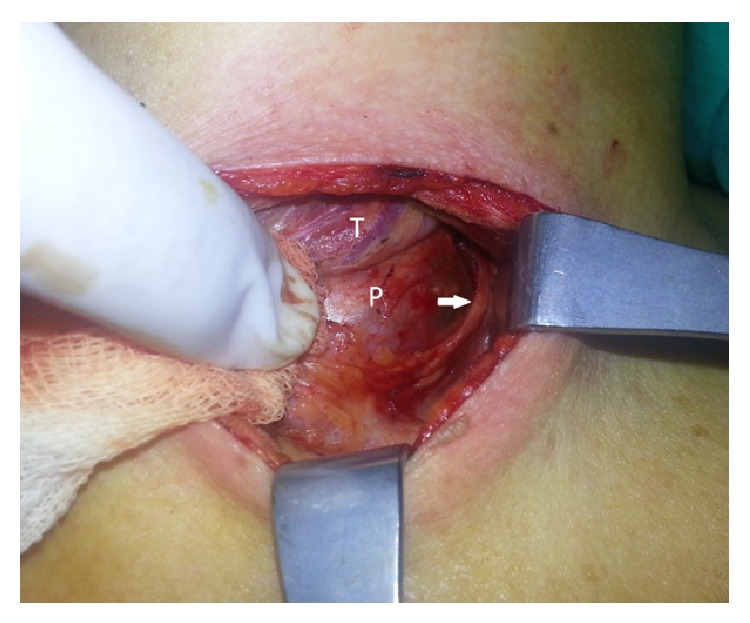
Left lobectomy and parathyroid adenomectomy. T: thyroid; P: parathyroid; arrow indicates the arterial shift caused by cyst.

**Figure 4:**
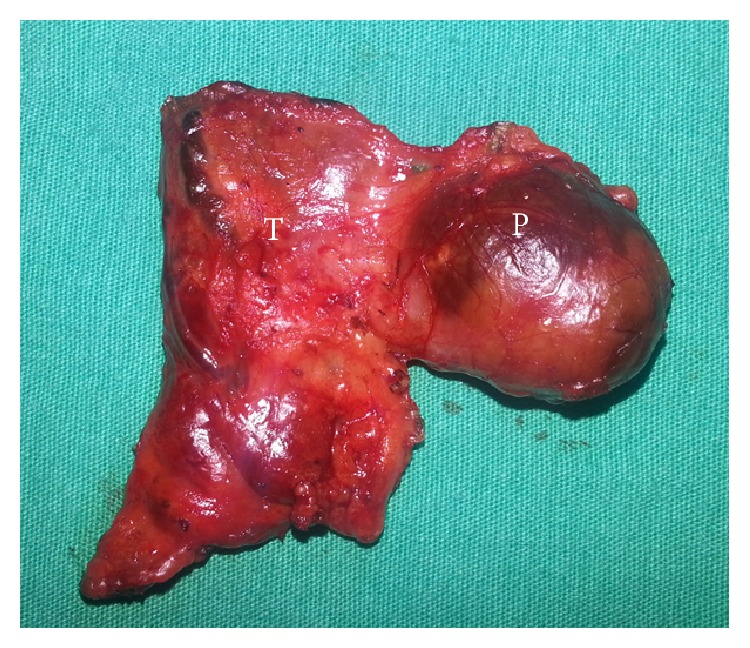
Macroscopic image of left thyroid lobe and parathyroid adenoma.

Fine needle aspiration (FNA) from cystic thyroid nodule revealed many macrophages and histiocytes with a few degenerative changes over an eosinophilic-fibrinous background. iPTH level was measured from the cyst aspiration fluid and it was higher (iPTH > 600 pg/mL) than serum iPTH level. The patient was diagnosed with primary hyperparathyroidism due to functional parathyroid cyst and left lobectomy and excision of left parathyroid adenomectomy was performed (Figures 3 and 4). In exploration, other parathyroid glands were observed as normal.

**Figure 5:**
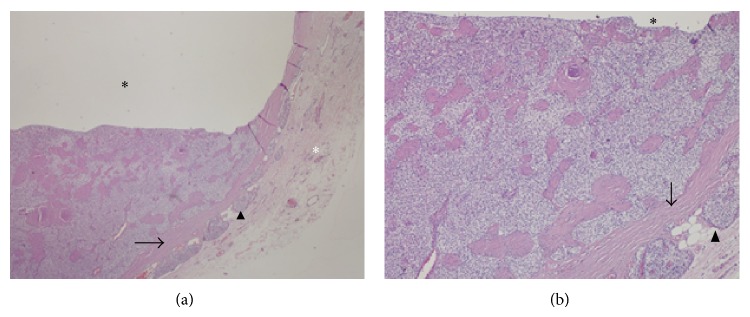
Parathyroid adenoma presenting cystic degeneration. Cystic lesion consisting of parathyroid cells separated from parathyroid gland with fibrous capsule. Note that the parathyroid parenchyma is squeezed in peripheral region of the lesion ((a) H&E ×40, (b) H&E ×100, black asterisk; cystic space, white asterisk; extraparathyroidal area, arrow; fibrous capsule, arrow head; squeezed parathyroid tissue).

Histopathological evaluation showed apparently encapsulated parathyroid adenoma which had a cystic center. Adenoma consisted mainly of chief cells and less frequently of water-clear cells. Cells forming the adenoma were located in a trabecular and solid fashion. There was no adipose tissue in adenoma. Outside the capsule, compressed parathyroid parenchyma was seen (Figure 5). Serum Ca decreased to 8.5 mg/dL and iPTH decreased to 1.7 pg/mL postoperatively. Follow-up visits were arranged for the patient.

## 3. Discussion

Primary hyperparathyroidism is a relatively common disease. However parathyroid cysts are rare and approximately 300 cases have been reported in the literature. They form 0.08–3.41% of all parathyroid masses [8]. However intrathyroidal parathyroid cysts are very rare conditions with only a few cases that have been reported so far [9–12]. Most of the parathyroid cysts are nonfunctional. Functional cysts are generally thought to be due to cystic degeneration of parathyroid adenomas. In the presented case, we detected a rare functional intrathyroidal parathyroid cyst.

Imaging methods like Tc^99m^ sestamibi and thyroid ultrasonography which are used for detecting localisation of parathyroid adenoma may not differentiate between thyroid and parathyroid cysts. Similarly, in this case, although there were laboratory findings implying primary hyperparathyroidism, no lesion consistent with parathyroid adenoma was detected in thyroid ultrasonography. Instead, a lesion suggesting a localised thyroid cyst was reported in left thyroid lobe. No radioactive material uptake was seen in Tc^99m^ sestamibi imaging. In this case iPTH measurement in cyst aspiration fluid obtained by fine needle aspiration is helpful. A higher iPTH level in cyst fluid than serum is diagnostic. Increased iPTH level in cyst fluid differentiates parathyroid cyst and thyroid cyst [2, 7, 13]. In our case, diagnosis of functional parathyroid cyst was made according to the higher iPTH level measured in aspiration fluid from the cyst, compared to serum iPTH level.

Optimal treatment for symptomatic or functional parathyroid cysts is surgical resection. In the presented case, serum iPTH level was decreased >50% and serum Ca level was normalized after excision of cystic parathyroid lesion with left lobectomy. Histopathological evaluation revealed apparently encapsulated parathyroid adenoma with a cystic center and this finding confirmed that intrathyroidally located cystic parathyroid adenoma was responsible for primary hyperparathyroidism.

First report of a functional intrathyroidal parathyroid cyst in the literature is a 54-year-old male patient presenting with nephrolithiasis. Tc^99m^ sestamibi parathyroid scintigraphy showed an uptake pattern consistent with parathyroid adenoma in right lower pole of thyroid gland. Thyroid ultrasonography showed a 1.7 cm cystic nodule in right lower pole of thyroid gland. Sample fluid from the cyst was not sufficient for iPTH analysis. After right hemithyroidectomy, serum Ca and iPTH levels normalised in that patient and histopathological evaluation was consistent with a parathyroid cyst at inferior pole [9].

A study measured thyroglobulin, PTH, and calcitonin levels from fluid samples which were obtained by fine needle aspirations from 112 patients detected to have cystic lesions in neck. Seven cases (6,2%) were diagnosed with incidental parathyroid cysts [7].

Our case was with an intrathyroidal functional parathyroid cystic adenoma who presented with primary hyperparathyroidism. The diagnosis was made after aspiration of cyst fluid and detection of increased PTH concentration in this fluid. Optimal treatment for functional parathyroid cysts is surgery. Cure was achieved in our patient after surgical excision with left lobectomy.

## References

[1] Wermers R. A., Khosla S., Atkinson E. J. (2006). Incidence of primary hyperparathyroidism in Rochester, Minnesota, 1993–2001: an update on the changing epidemiology of the disease. *Journal of Bone and Mineral Research*.

[2] Ippolito G., Palazzo F. F., Sebag F., Sierra M., De Micco C., Henry J.-F. (2006). A single-institution 25-year review of true parathyroid cysts. *Langenbeck's Archives of Surgery*.

[3] Ihm P. S., Dray T., Sofferman R. A., Nathan M., Hardin N. J. (2001). Parathyroid cysts: diagnosis and management. *Laryngoscope*.

[4] Shields T. W., Immerman S. C. (1999). Mediastinal parathyroid cysts revisited. *Annals of Thoracic Surgery*.

[5] Fortson J. K., Patel V. G., Henderson V. J. (2001). Parathyroid cysts: a case report and review of the literature. *Laryngoscope*.

[6] Ujiki M. B., Nayar R., Sturgeon C., Angelos P. (2007). Parathyroid cyst: often mistaken for a thyroid cyst. *World Journal of Surgery*.

[7] Pacini F., Antonelli A., Lari R., Gasperini L., Baschieri L., Pinchera A. (1985). Unsuspected parathyroid cysts diagnosed by measurement of thyroglobulin and parathyroid hormone concentrations in fluid aspirates. *Annals of Internal Medicine*.

[8] Tamiya H., Miyakawa M., Suzuki H. (2013). A large functioning parathyroid cyst in a patient with multiple endocrine neoplasia type 1. *Endocrine Journal*.

[9] Rickels M. R., Langer J. E., Mandel S. J. (2004). Hyperfunctioning intrathyroidal parathyroid cyst. *The Journal of Clinical Endocrinology and Metabolism*.

[10] Capezzone M., Morabito E., Bellitti P., Giannasio P., de Santis D., Bruno R. (2007). Ectopic intrathyroidal nonfunctioning parathyroid cyst. *Endocrine Practice*.

[11] Atwan M., Chetty R. (2011). An unusual “thyroid cyst”: intrathyroidal parathyroid cyst. *Endocrine Pathology*.

[12] Dutta D., Selvan C., Kumar M. (2013). Needle aspirate PTH in diagnosis of primary hyperparathyroidism due to intrathyroidal parathyroid cyst. *Endocrinology, Diabetes and Metabolism Case Reports*.

[13] Abraham D., Sharma P. K., Bentz J., Gault P. M., Neumayer L., McClain D. A. (2007). Utility of ultrasound-guided fine-needle aspiration of parathyroid adenomas for localization before minimally invasive parathyroidectomy. *Endocrine Practice*.

